# Global Trends in Research of Programmed Cell Death in Osteoporosis: A Bibliometric and Visualized Analysis (2000–2023)

**DOI:** 10.1111/os.14133

**Published:** 2024-06-24

**Authors:** Yi‐fa Rong, Xue‐Zhen Liang, Kai Jiang, Hai‐Feng Jia, Han‐Zheng Li, Bo‐Wen Lu, Gang Li

**Affiliations:** ^1^ The First College of Clinical Medicine Shandong University of Traditional Chinese Medicine Jinan China; ^2^ Orthopaedic Microsurgery Affiliated Hospital of Shandong University of Traditional Chinese Medicine Jinan China

**Keywords:** Autophagy, Bibliometrix, Osteoporosis, Oxidative Stress, Programmed Cell Death

## Abstract

Osteoporosis (OP) is a systemic metabolic bone disease that is characterized by decreased bone mineral density and microstructural damage to bone tissue. Recent studies have demonstrated significant advances in the research of programmed cell death (PCD) in OP. However, there is no bibliometric analysis in this research field. This study searched the Web of Science Core Collection (WoSCC) database for literature related to OP and PCD from 2000 to 2023. This study used VOSviewers 1.6.20, the “bibliometrix” R package, and CiteSpace (6.2.R3) for bibliometric and visualization analysis. A total of 2905 articles from 80 countries were included, with China and the United States leading the way. The number of publications related to PCD in OP is increasing year by year. The main research institutions are Shanghai Jiao Tong University, Chinese Medical University, Southern Medical University, Zhejiang University, and Soochow University. Bone is the most popular journal in the field of PCD in OP, and the Journal of Bone and Mineral Research is the most co‐cited journal. These publications come from 14,801 authors, with Liu Zong‐Ping, Yang Lei, Manolagas Stavros C, Zhang Wei, and Zhao Hong‐Yan having published the most papers. Ronald S. Weinstein was co‐cited most often. Oxidative stress and autophagy are the current research hot spots for PCD in OP. This bibliometric study provides the first comprehensive summary of trends and developments in PCD research in OP. This information identifies the most recent research frontiers and hot directions, which will provide a definitive reference for scholars studying PCD in OP.

## Graphical abstract

In this study, 2905 studies in the field of programmed cell death in osteoporosis research in Web of Science database from 2000 to 2023 were analyzed qualitatively and quantitatively using bibliometric software like CiteSpace, VOSviwer, and the “bibliometrix” R package. This study provides new insights into basic and applied research in this field and provides researchers with a valuable resource so they can comprehensively assess their research area and discover new research directions.

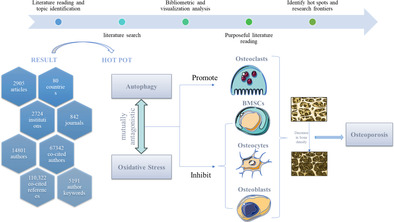

## Introduction

Osteoporosis (OP) is a systemic metabolic bone disease characterized by decreased bone density and damage to the microstructure of bone tissue, leading to increased bone fragility and risk of fracture.^1^ With the global population aging, osteoporosis affects over 200 million people, making it a significant public health threat worldwide.^2^, ^3^ The economic burden of this disease is growing rapidly, with osteoporosis now costing $6.5 trillion annually in the United States, Canada, and Europe alone, yet most people are unaware of the dangers of this insidious disease.^4^ Osteoporosis is primarily caused by an imbalance between bone formation and bone resorption, reducing bone mass and an increased risk of fractures.^1^ While there are numerous clinical therapeutic medications available for osteoporosis that can effectively restore bone strength, it is important to note that these medications may unintentionally reduce bone tone. Additionally, some of these medications can be expensive, have serious side effects, and require long‐term use.^5^ The search for new therapeutic mechanisms related to osteoporosis has received increasing attention from scientists.

Programmed cell death (PCD) is the process by which cell death occurs to maintain homeostasis in the internal environment after receiving certain signals or being subjected to certain stimuli.^6^, ^7^ Previous research has demonstrated that PCD plays an important role in regulating bone metabolism by affecting the activity of osteoblasts.^8^ In the past two decades, PCD has received significant attention, and its forms include cognitive apoptosis, necrotizing apoptosis, autophagy, pyroptosis, ferroptosis, and cuproptosis, and the forms are correlated with each other.^9^, ^10^ The in‐depth study of PCD can help to elucidate the molecular mechanism of OP‐associated osteocyte death, reveal its role in the pathogenesis of OP, and provide a new direction for the study of the therapeutic mechanism of OP.

Bibliometrics is a method of literature analysis that quantitatively and qualitatively analyzes the distribution structure, quantitative relationships, and patterns of change of relevant information in the literature to summarize the current status and development trends of a research field or disease, and to provide guidance for future development.^11^ We can extract detailed information such as keywords, authors, countries, institutions, journals, references in related research areas through bibliometric analysis.^12^ In addition, bibliometric tools such as CiteSpace,^13^ VoSviewer,^14^ and the R package “bibliometrix”^15^ have been used for visual literature analysis, as they are more intuitive for interpreting a large amount of information and mining research hotspots. Bibliometrics has been widely used in the fields of cancer,^16^ knee osteoarthritis,^17^ cardiovascular diseases,^18^ and rheumatic diseases,^19^ but no bibliometric method has been used to research PCD in OP. Therefore, to fill this knowledge gap, this study used bibliometrics and visual analytics to summarize the academic research on PCD in OP over the last two decades (2000–2023) in the Web of Science database. The study aims to identify the main contributors and the current state of research in the field of PCD in OP, and to explore the global research hotspots and development trends in order to provide relevant guidance to researchers in the field.

## Methods

### Search Strategy

**FIGURE 1:**
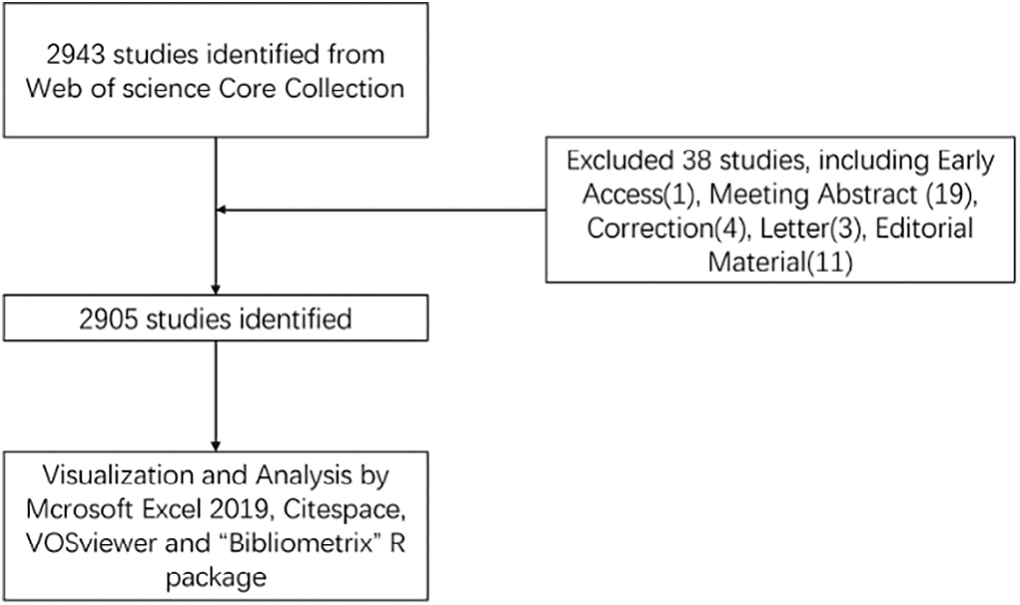
Publications screening flowchart.

We conducted a literature search related to OP and PCD in the Web of Science Core Collection (WoSCC) database on February 11, 2024. Our search strategy was as follows: The research formula is (TS = (Osteoporosis)) AND ((((((TS = (programmed cell death)) OR TS = (apoptosis)) OR TS = (pyroptosis)) OR TS = (autophagy)) OR TS = (ferroptosis)) OR TS = (cuproptosis)). The language limit was English, the time period was set from January 1, 2000 to December 31, 2023, and the type of document was set to “articles” and “review” (Figure 1).

### Data Analysis

VOSviewer (version 1.6.20, https://www.vosviewer.com/) is a bibliometric analysis software that provides text mining capabilities for building and visualizing co‐occurrence networks of important terms extracted from scientific literature.^20^, ^21^ In this study, the software mainly completed the following analyses: country and institution analysis, journal and co‐cited journal analysis, author and co‐cited author analysis, and keyword co‐occurrence analysis. In the visualization network generated by VOSviewer, different nodes represent different components such as countries, institutions, journals, and authors. The size and color of the nodes indicate the number and classification of these items, respectively. Links between nodes represent co‐occurrence relationships, and the size of a link indicates the frequency of co‐occurrence of two nodes.

CiteSpace (version 6.2.R3) is a bibliometric analysis and visualization software developed by Professor C. Chen.^22^, ^23^ It can be used to explore trends and dynamics of scientific research in specific research areas. In this study, CiteSpace was applied to map the dual‐map overlay of journal and analyze reference with citation bursts to identify emerging themes.

The R package “bibliometrix” (version 4.3.1) (https://www.bibliometrix.org) allows bibliometric analysis of relevant literature.^24^ In this study, the R package “bibliometrix” is mainly used to analyze the thematic evolution and the global distribution network of publications. The quartile and impact factors of the journals were taken from Journal Citation Reports 2022. In addition, the publications were quantitatively analyzed using Microsoft Office Excel 2019.

## Result

### Quantitative Analysis of Publications

**FIGURE 2:**
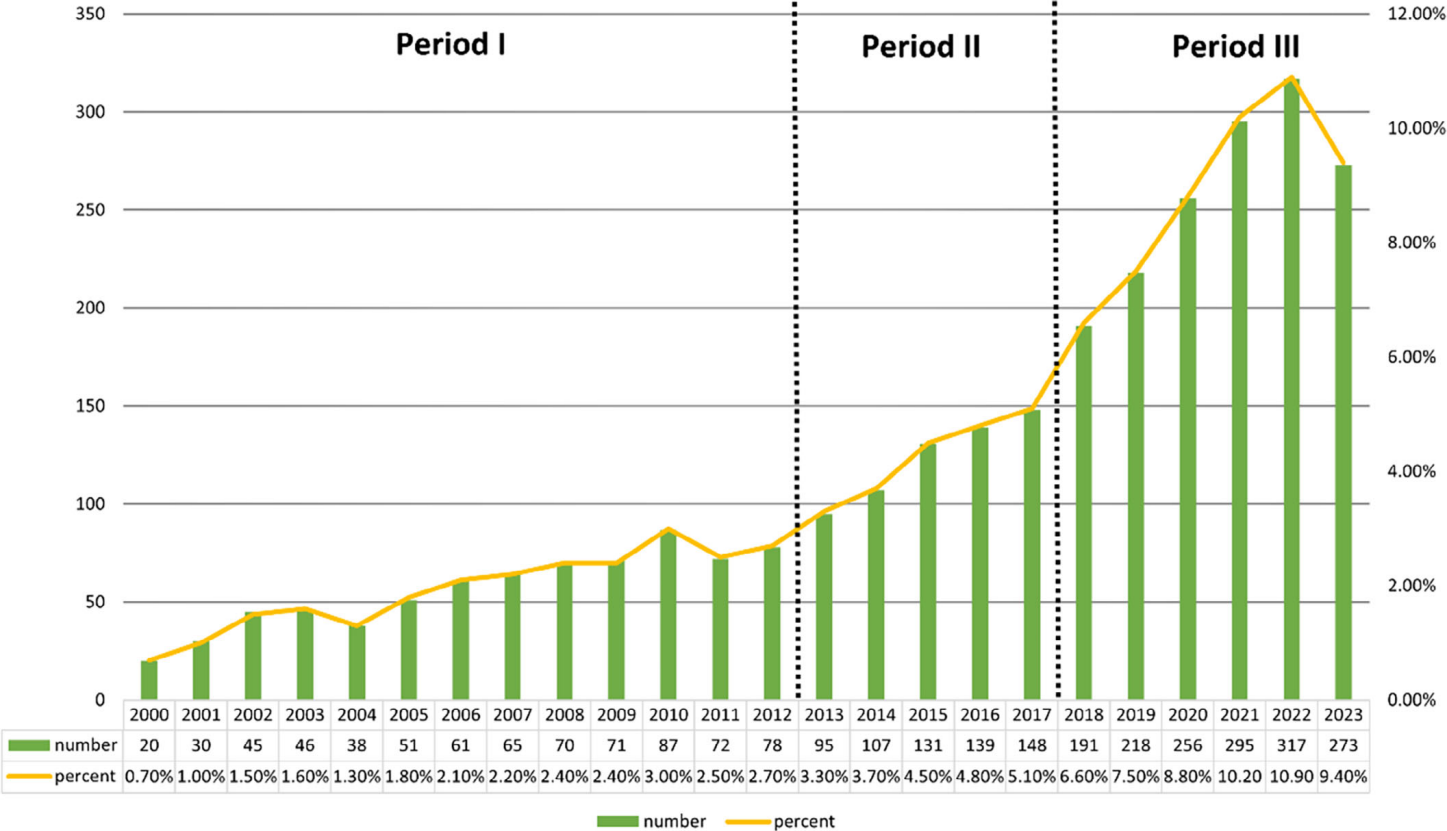
Annual output of research of PCD in OP.

According to our search results, there were 2905 studies of OP in PCD in the past two decades, including 2361 “articles” and 544 “reviews.” Judging from the growth rate of the number of publications each year, the whole period can be divided into three parts: Period I (2000–2012), Period II (2013–2017), and Period III (2018–2023). As shown in Figure 2, the number of publications in Period I is relatively small, with an average annual publication number of about 60, which is in the initial stage of the research of PCD in OP. The number of papers published in Period II increased, with an average annual number of about 120, and represents the development stage of PCD and osteoporosis research. The number of publications in Period III began to increase significantly, and represents the maturation period of PCD and OP studies. The annual average number of publications is about 240, and the total number of papers in this stage is significantly higher than that in the other two stages.

### Analysis of Countries and Institution

**FIGURE 3:**
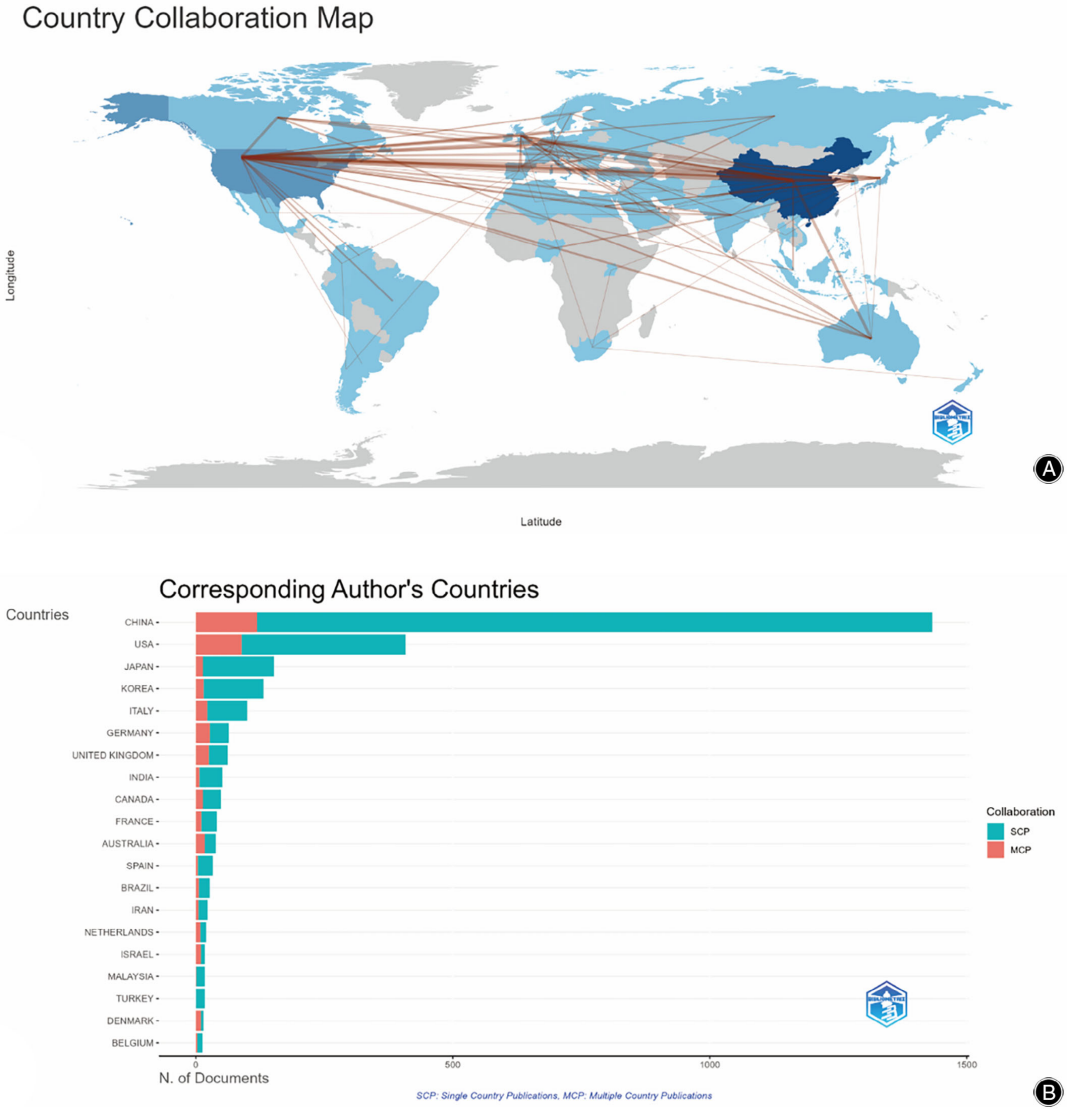
The geographical distribution on research of PCD in OP (A). Top 20 countries in terms of number of corresponding authors (B).

**FIGURE 4:**
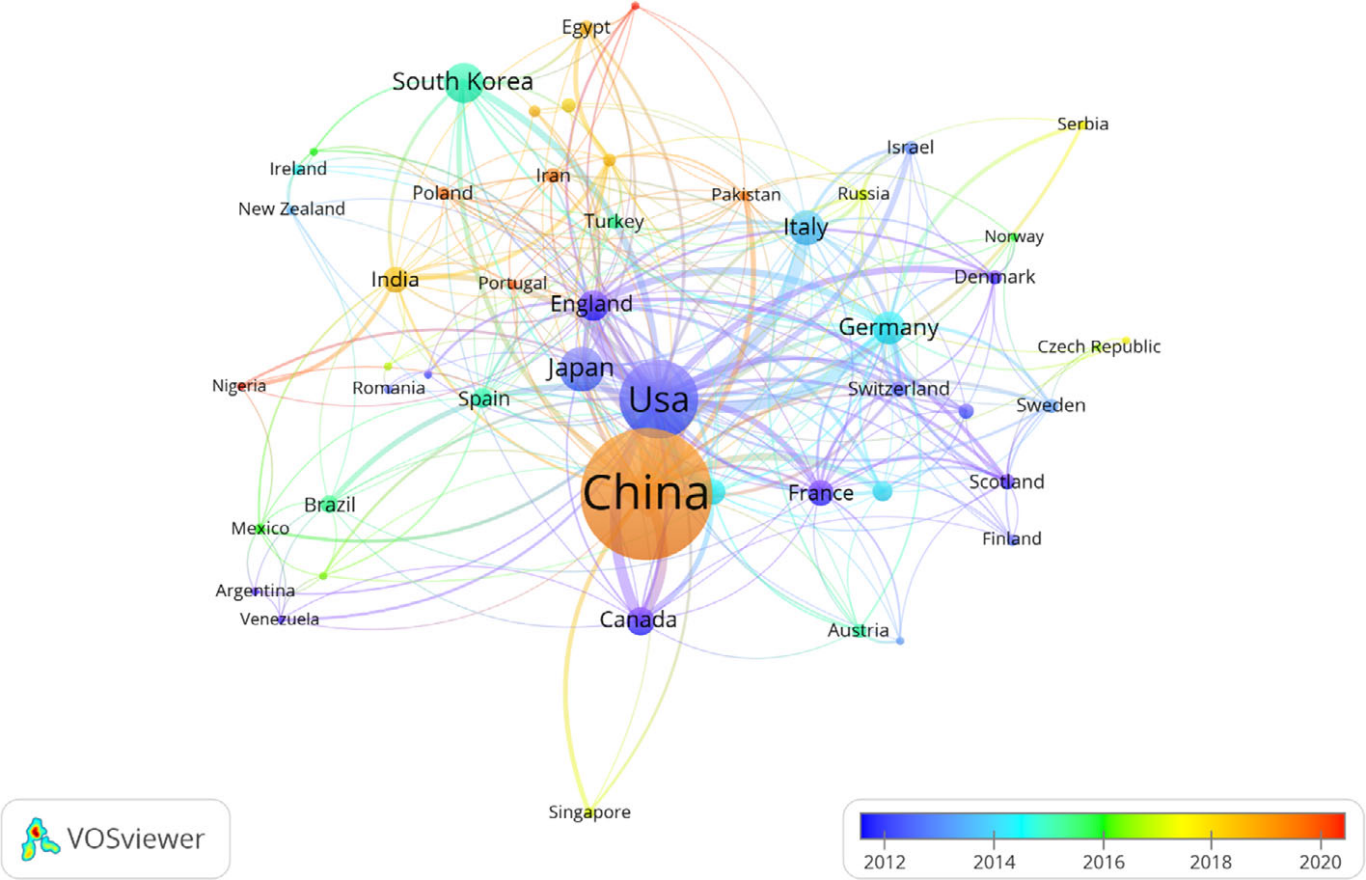
Visualization of countries on research of PCD in OP.

These publications were from 80 countries and 2724 institutions. The top 10 countries were China, the United States, Japan, South Korea, Italy, Germany, England, Canada, Australia, and India, which four in Asia, three in Europe, two in North America, and one in Oceania (Table 1). Among the countries, the one with the largest number of publications was China (n = 1482, 51.0%), followed by the United States (n = 544, 18.7%), Japan (n = 179, 6.2%), South Korea (n = 148, 5.1%), and Italy (n = 117, 4.0%). The combined number of publications from China and the United States accounted for almost half of the total (69.7%). Subsequently, we filtered and visualized 50 countries that had a number of publications greater than or equal to five and constructed a collaborative network based on the number and relationship of publications in each country (Figure 3B). As shown in Figures 3A and 4, The thicker the link between the two countries, the more cooperation there is between them. Notably, there is much active cooperation between different countries. For example, China has close cooperation with the United States (77), South Korea (7), Singapore (6), Sweden (4), and Switzerland (4). Italy has a active cooperation with the United States (22). Canada has an active cooperation with Serbia (5). In Figure 3B, SCP indicates articles whose authors are all from the same country, while MCP indicates articles whose authors are from multiple countries, indicating international collaboration. Therefore, the study inferred that China has the most co‐authors in this field with other countries, but the collaboration within China is greater.

**TABLE 1:** Top 10 countries and institutions on research of PCD in OP.

Rank	Country	Counts	Percent (%)	Institution	Counts	Percent (%)
1	China (Asia)	1482	51.0	Shanghai jiao tong univ	76	2.6
2	United States (North America)	544	18.7	China med univ	69	2.4
3	Japan (Asia)	179	6.2	Southern med univ	52	1.8
4	South Korea (Asia)	148	5.1	Zhejiang univ	52	1.8
5	Italy (Europe)	117	4.0	Soochow univ	51	1.8
6	Germany (Europe)	98	3.4	Nanjing med univ	48	1.7
7	England (Europe)	88	3.0	Univ Arkansas Med Sci	43	1.5
8	Canada (North America)	77	2.7	Xi an jiao tong univ	41	1.4
9	Australia (Oceania)	66	2.3	Fourth mil med univ	38	1.3
10	India (Asia)	65	2.2	Sichuan univ	38	1.3

**FIGURE 5:**
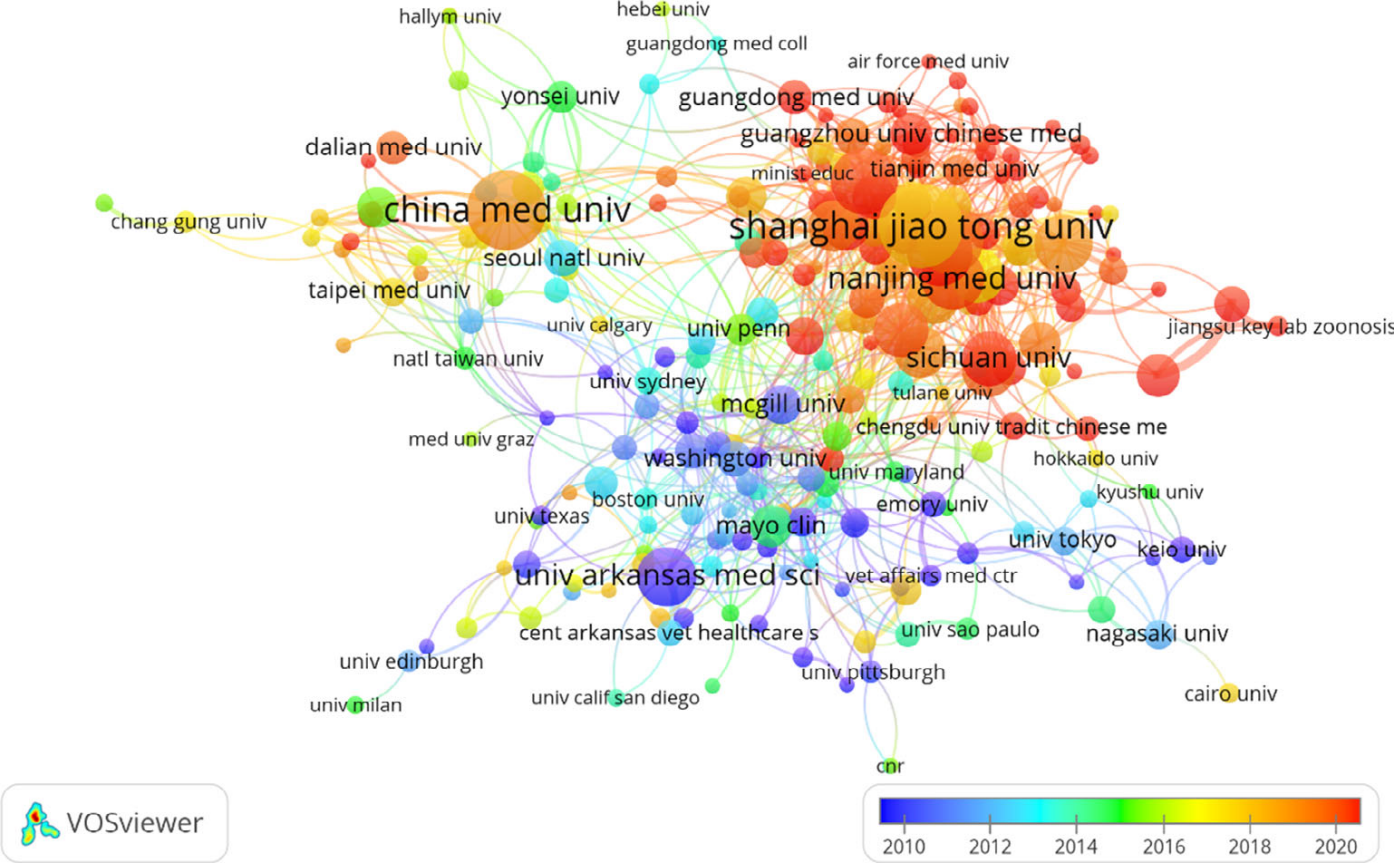
The visualization of institutions on research of PCD in OP.

Nine of the top 10 institutions are located in China, with the remaining one in the United States. The four institutions that published the most relevant papers are Shanghai Jiao Tong University (n = 76, 2.62%), Chinese Medical University (n = 69, 2.38%), Southern Medical University (n = 52, 1.79%), Zhejiang University (n = 52, 1.79%), and Soochow University (n = 51, 1.76%) (Table 1). Subsequently, we selected 273 institutions that had a minimum number of publications equal to five for visualization and constructed a collaborative network based on the number and relationship of publications of each institution (Figure 5). As shown in Figure 5, we note that the research institutions are mostly universities from various countries and Shanghai Jiao Tong University has published the largest number of papers and has close partnerships with other institutions, such as Fudan University, Zhejiang University, Tongji University, the Chinese Academy of Sciences, and the Second Military Medical University.

### Journals and Co‐Cited Journals

**FIGURE 6:**
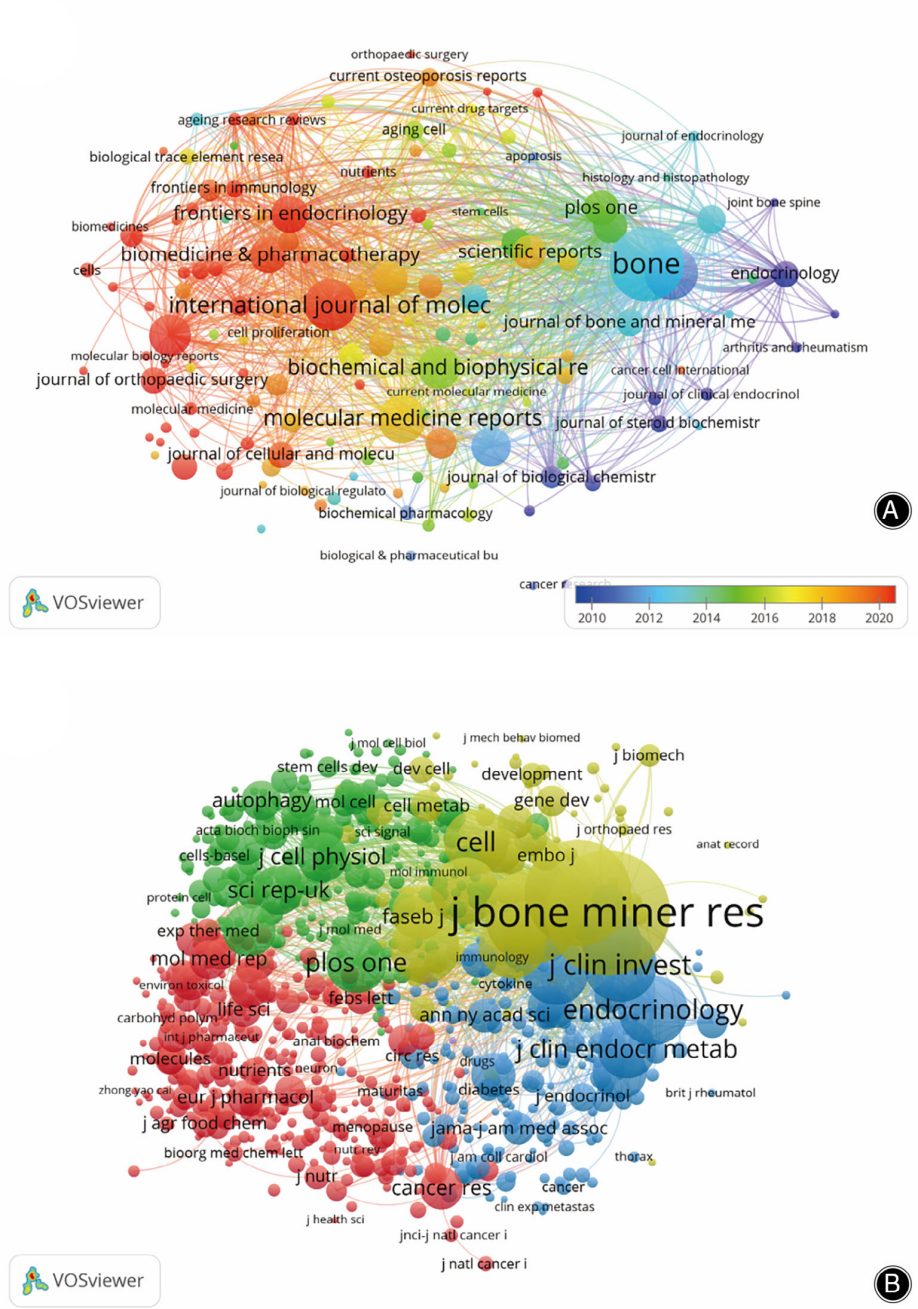
The visualization of journals (A) and co‐cited journals (B) on research of PCD in OP.

Publications related to PCD in OP were published in 842 journals. *Bone* published the most papers (n = 101, 3.5%), followed by *Journal of Bone and Mineral Research* (n = 63, 2.2%), *International Journal of Molecular Sciences* (n = 60, 2.1%), *Molecular Medicine Reports* (n = 54, 1.9%), *Biochemical and Biophysical Research Communications* (n = 46, 1.6%). Among the top 20 journals, there were six in Q1 and 10 in Q2 in Journal Citation Reports (JCR), and the journal with the highest impact factor was *Biomedicine & Pharmacotherapy* (IF = 7.5), followed by *Journal of Bone and Mineral Research* (IF = 6.2) and *Frontiers in Pharmacology* (IF = 5.6) (Table 2). Subsequently, we screened all journals based on the minimum number of relevant publications equal to five and mapped the journal network (Figure 6A). Figure 6A shows that *Bone* has active citation relationships with *Journal of Bone and Mineral Research*, *Calcified Tissue International*, *Osteoporosis International*, *Endocrinology*, and others.

**TABLE 2:** Top 20 journals for research of PCD in OP.

Rank	Journal	Count	IF	JCR
1	Bone	101 (3.5%)	4.1	Q1
2	Journal of Bone and Mineral Research	63 (2.2%)	6.2	Q1
3	International Journal of Molecular Sciences	60 (2.1%)	5.6	Q1
4	Molecular Medicine Reports	54 (1.9%)	3.4	Q3
5	Biochemical and Biophysical Research Communications	46 (1.6%)	3.1	Q3
6	Frontiers in Pharmacology	45 (1.5%)	5.6	Q1
7	Journal of Cellular Biochemistry	41 (1.4%)	4.0	Q2
8	Frontiers in Endocrinology	38 (1.3%)	5.2	Q1
9	Biomedicine & Pharmacotherapy	37 (1.3%)	7.5	Q1
10	Journal of Cellular Physiology	34 (1.2%)	5.6	Q2
11	Plos One	34 (1.2%)	3.7	Q2
12	Calcified Tissue International	33 (1.1%)	4.2	Q2
13	Scientific Reports	32 (1.1%)	4.6	Q2
14	Experimental and Therapeutic Medicine	29 (1.0%)	2.7	Q3
15	International Journal of Molecular Medicine	27 (0.9%)	5.4	Q2
16	Osteoporosis International	26 (0.9%)	4.0	Q2
17	European Journal of Pharmacology	24 (0.8%)	5.0	Q1
18	Journal of Bone and Mineral Metabolism	24 (0.8%)	3.3	Q3
19	Endocrinology	23 (0.8%)	4.9	Q2
20	Journal of Cellular and Molecular Medicine	23 (0.8%)	5.3	Q2

As shown in Table 3, among the top 20 co‐cited journals, three journals were co‐cited more than 3000 times: *Journal of Bone and Mineral Research* (co‐citations = 7682) was the most co‐cited journal, followed by *Bone* (co‐citations = 5546) and *Journal of Biological Chemistry* (co‐citations = 4423). In addition, the impact factor of the *New England Journal of Medicine* is the highest (IF = 158.5), followed by *Nature Medicine* (IF = 82.9) and *Nature* (IF = 64.8). Journals with a minimum co‐citation equal to 25 were filtered to map the co‐citation network (Figure 6B). As shown in Figure 6B, *Bone* has positive co‐citation relationships with *Journal of Bone and Mineral Research, Journal of Biological Chemistry*, *Endocrinology* and *Journal of Clinical Investigation*.

**TABLE 3:** Top 20 co‐cited journals for research of PCD in OP.

Rank	Journal	Co‐citation	IF	JCR
1	Journal of Bone and Mineral Research	7683	6.2	Q1
2	Bone	5546	4.1	Q1
3	Journal of Biological Chemistry	4425	4.8	Q2
4	Journal of Clinical Investigation	2612	15.9	Q1
5	Biochemical and Biophysical Research Communications	2413	3.1	Q3
6	Endocrinology	2413	4.9	Q2
7	Proceedings of the National Academy of Sciences of the United States of America	2228	11.1	Q1
8	Nature	2196	64.8	Q1
9	Osteoporosis International	2093	4.0	Q2
10	Cell	2081	64.5	Q1
11	Plos One	1839	3.7	Q2
12	Calcified Tissue International	1781	4.2	Q2
13	Journal of Clinical Endocrinology & Metabolism	1697	5.8	Q1
14	Journal of Cellular Biochemistry	1528	4.0	Q2
15	Science	1498	56.9	Q1
16	New England Journal of Medicine	1486	158.5	Q1
17	International Journal of Molecular Sciences	1400	5.6	Q1
18	Journal of Cellular Physiology	1217	5.6	Q2
19	Scientific Reports	1172	4.6	Q2
20	Nature Medicine	988	82.9	Q1

**FIGURE 7:**
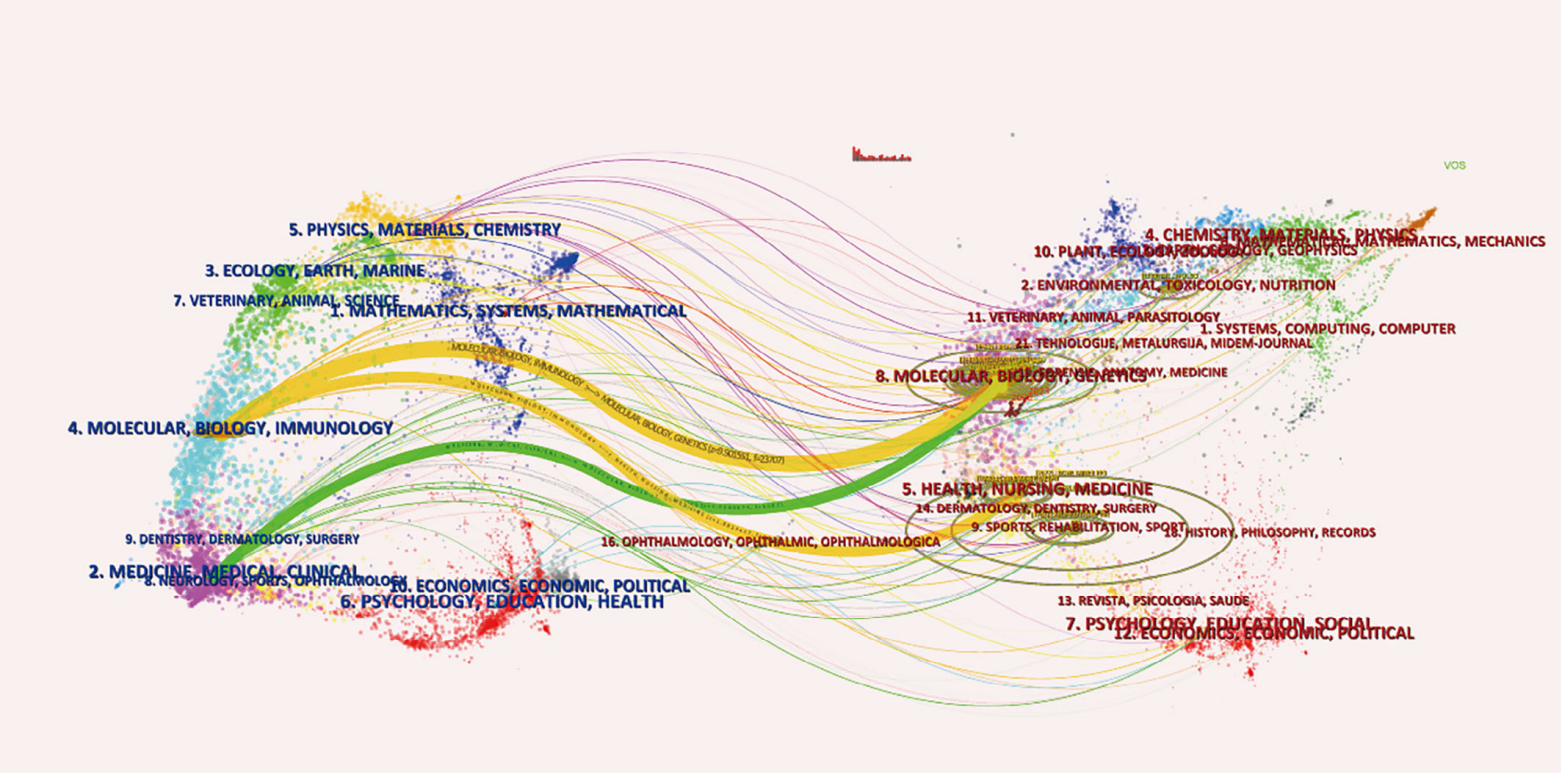
Dual‐map overlay of journals on research of PCD in OP.

The dual‐map overlay of journals shows the citation relationships between journals and co‐cited journals, with clusters of citing journals on the left and clusters of cited journals on the right. As shown in Figure 7, the orange and green path are the main citation paths. The orange path represents the research published in four molecular, biology, immunology journals is mainly cited by the literature in eight molecular, biology, genetics journals and five health, nursing, medicine journals. The green path represents the research published in two medicine, medical clinical journals is mainly cited in the literature in eight molecular, biology, genetics journals.

### Author and Co‐Cited Author

**FIGURE 8:**
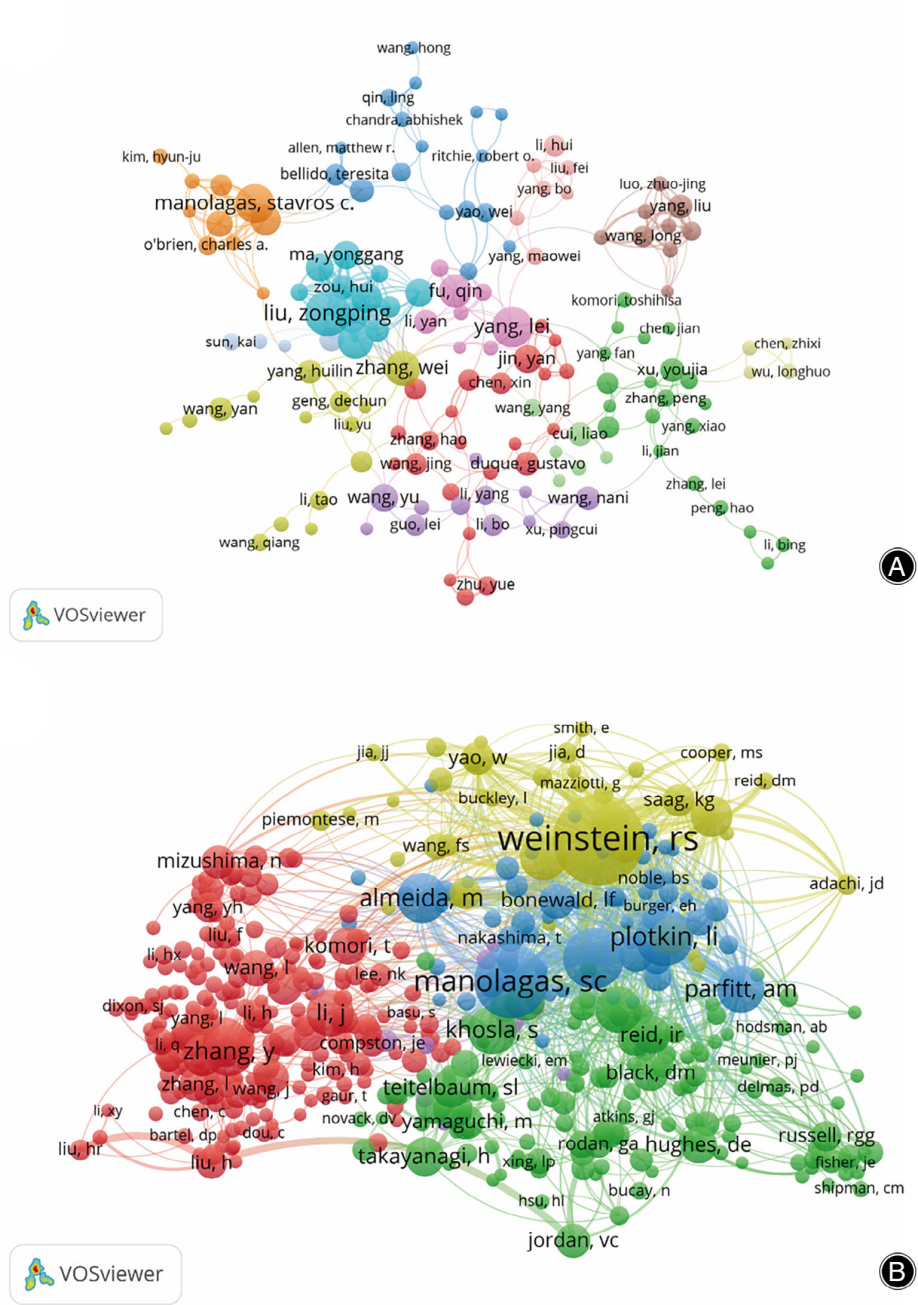
Visualization of authors (A) and co‐cited authors (B) on research of PCD in OP.

A total of 14,801 authors participated in PCD research in OP. Among the top 20 authors, each author published no less than 10 papers (Table 4). We built a collaborative network based on authors whose number of published papers was greater than or equal to five (Figure 8A). Liu Zong‐Ping, Yang Lei, Stavros C. Manolagas, Zhang Wei, and Zhao Hong‐Yan had larger nodes because they had more related publications. In addition, we observed close collaboration among multiple authors. For example, Liu Zong‐Ping had close cooperation with Ma Yong‐Gang, Ran Di, Song Rui‐Long.

**TABLE 4:** Top 20 authors and co‐cited authors on research of PCD in OP.

Rank	Author	Count	Co‐cited authors	Citations
1	Liu, Zongping	20	Weinstein, RS	743
2	Yang, Lei	18	Manolagas, SC	521
3	Manolagas, Stavros C	17	Jilka, RL	382
4	Zhang, Wei	15	Plotkin, Li	349
5	Zhao, Hongyan	15	Zhang, Y	271
6	Fu, Qin	14	Almeida, M	269
7	Bian, Jianchun	13	Khosla, S	263
8	Gu, Jianhong	13	Li, J	251
9	Weinstein, Robert S	13	Parfitt, AM	251
10	Jin, Yan	12	Canalis, E	247
11	Liu, Wei	12	Hofbauer, LC	242
12	Ma, Yonggang	12	Wang, Y	220
13	Song, Ruilong	12	Van Staa, TP	205
14	Wang, Yu	12	Chen, X	197
15	Almeida, Maria	11	Lane, NE	187
16	Cui, Liao	10	Takayanagi, H	186
17	Duque, Gustavo	10	Kanis, JA	185
18	Li, Yan	10	Teitelbaum, SL	185
19	Plotkin, Lilian I	10	Boyle, WJ	183
20	Ran, Di	10	Li, Y	175

Among the 67,342 co‐cited authors, 13 authors were co‐cited more than 200 times (Table 4). The most frequently co‐cited author is Robert S. Weinstein (n = 743), followed by Stavros C. Manolagas (n = 521), and Robert L. Jilka, (n = 382). Authors with minimum co‐citations equal to 30 were filtered to map co‐citation network graphs (Figure 8B). As shown in Figure 8B, there are also active collaborations among different co‐cited authors, such as Ronald S. Weinstein, Ma Yong‐Gang, Ran Di, and Song Rui‐Long.

### Cited References

**FIGURE 9:**
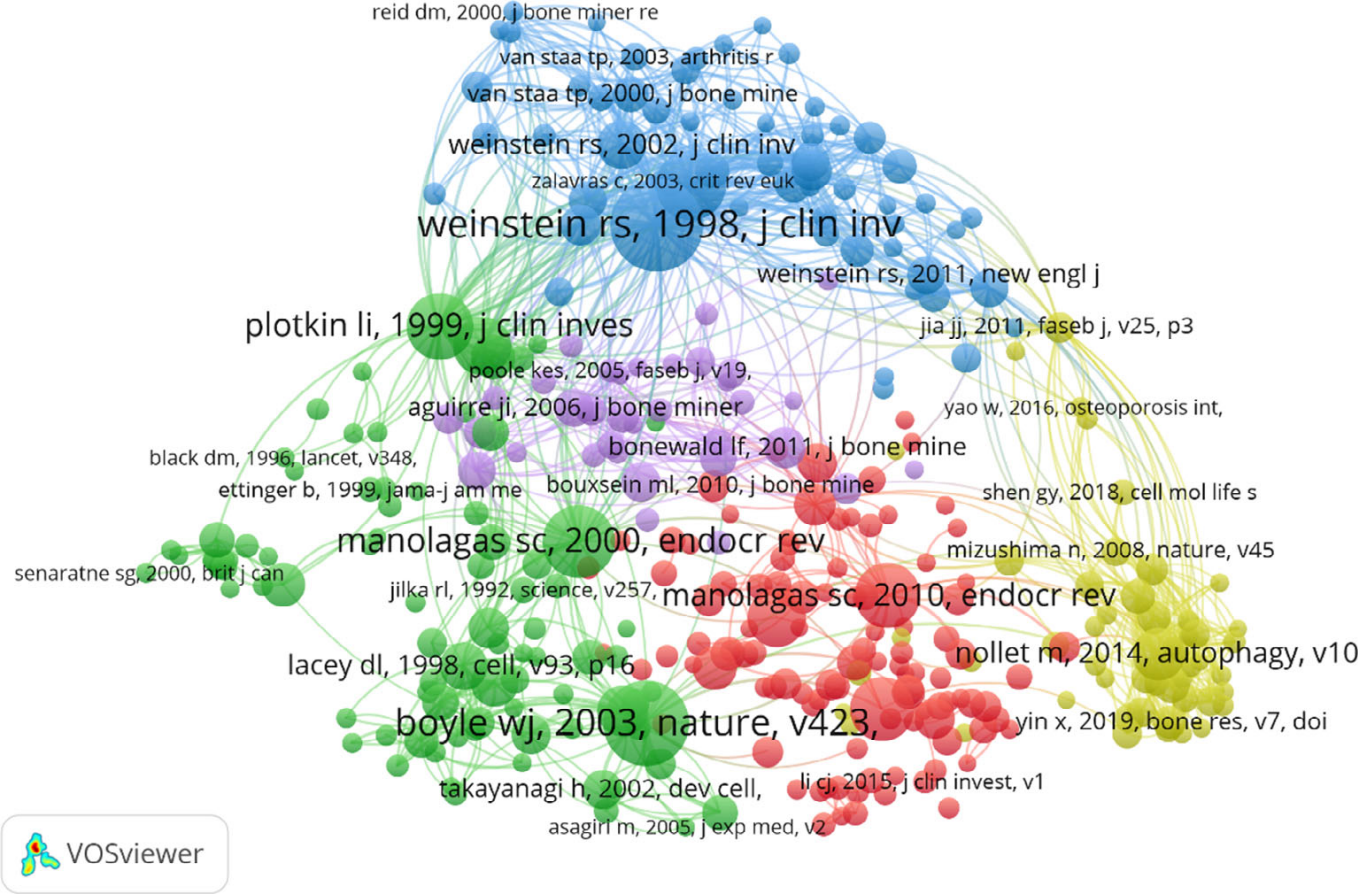
Visualization of co‐cited references on research of PCD in OP.

There have been 110,322 co‐cited references on PCD research in OP over the past two decades. In the top 20 co‐cited references (Table 5), all references were co‐cited at least 63 times, and five references were co‐cited more than 125 times. We selected references with co‐citations greater than or equal to 20 for the construction of the co‐citation network map (Figure 9). According to Figure 9, “weinstein rs, 1998, j clin invest” shows active co‐cited relationships with “weinstein rs, 2002, j clin invest,” “weinstein rs, 2000, j clin endocr metab,” “yao w, 2008, arthritis rheum,” and “weinstein rs, 2011, new engl j med.”

**TABLE 5:** Top 20 co‐cited references on research of PCD in OP.

Rank	Cited reference	Citations
1	Weinstein rs, 1998, j clin invest^25^	204
2	Boyle wj, 2003, nature^26^	181
3	Manolagas sc, 2000, endocr rev^27^	142
4	O'brien ca, 2004, endocrinology^28^	137
5	Plotkin li, 1999, j clin invest^29^	127
6	Manolagas sc, 2010, endocr rev^30^	125
7	Rachner td, 2011, lancet^31^	121
8	Livak kj, 2001, methods^32^	106
9	Jilka rl,1999, j clin invest^33^	105
10	Nollet m, 2014, autophagy^34^	90
11	Teitelbaum sl, 2000, science^35^	86
12	Weinstein rs, 2002, j clin invest^36^	82
13	Lacey dl, 1998, cell^37^	79
14	Simonet ws, 1997, cell^38^	79
15	Parfitt am, 1987, j bone miner res^39^	72
16	Raisz lg, 2005, j clin invest^40^	71
17	Hughes de, 1995, j bone miner res^41^	70
18	Bonewald lf, 2011, j bone miner res^42^	66
19	Almeida m, 2007, j biol chem^43^	65
20	Canalis e, 2007, oetsoporosis int^44^	64

### References with Citation Bursts

**FIGURE 10:**
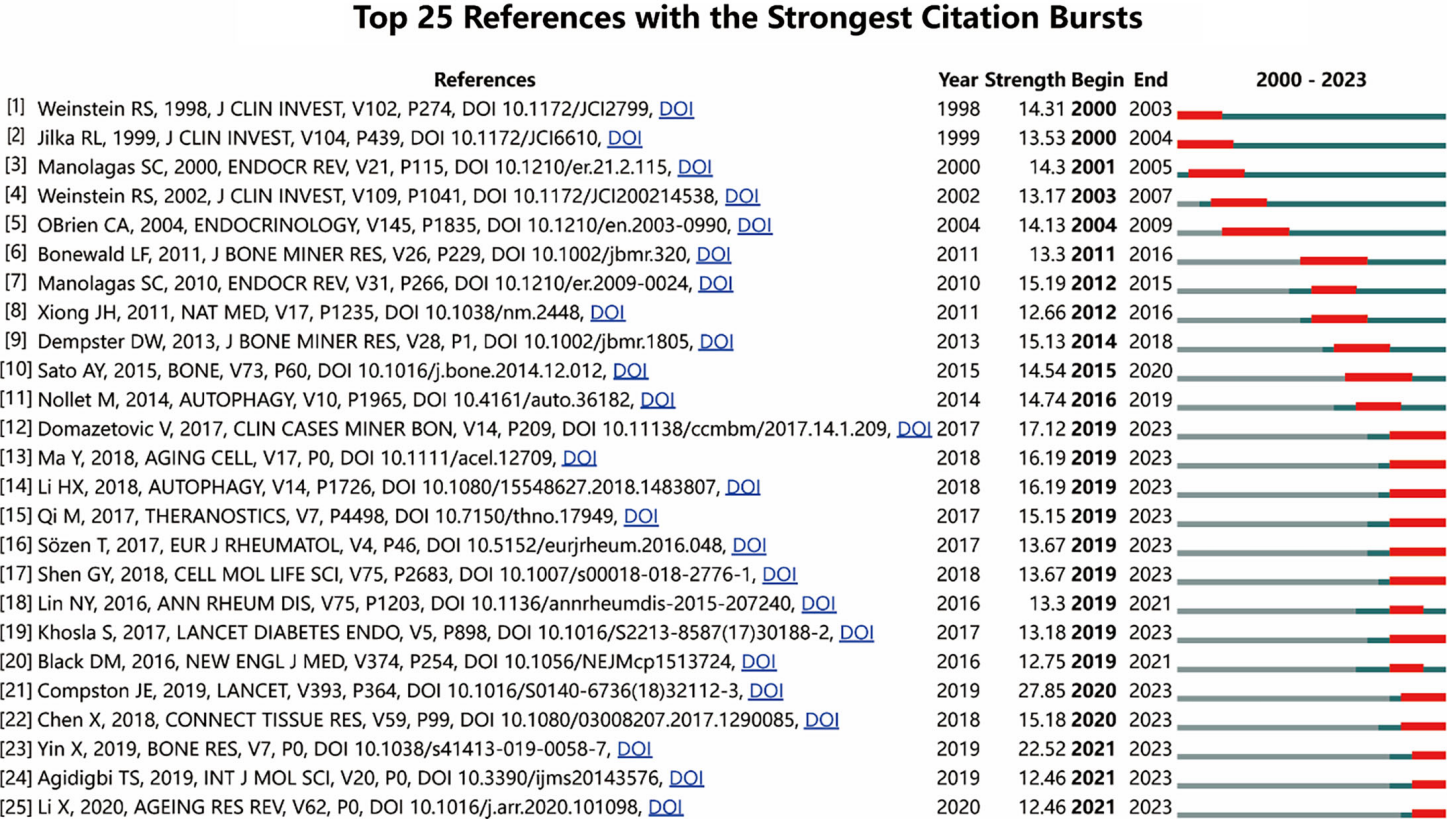
Top 25 references with strong citation bursts of PCD in OP. A red bar indicates high citations in that year.

References with citation bursts refer to those references that are frequently cited by scholars in a certain field over an interval of time. In our study, 25 references with strong citation bursts were identified by CiteSpace (Figure 10 and Table 6). As shown in Figure 10, every bar represents a year, and the red bar represents strong citation bursts. Citation bursts for references appeared as early as 2000 and as late as 2021. The reference with the strongest citation burst (strength = 27.84) was titled “Osteoporosis,” authored by J. E. Compston, with citation bursts from 2020 to 2023. The reference with the second strongest citation burst (strength = 22.52) was titled “Autophagy in bone homeostasis and the onset of osteoporosis,” authored by Yin Xing, with citation bursts from 2021 to 2023. Overall, the burst strength of these 25 references ranged from 12.46 to 27.84, and the endurance strength ranged from 3 to 6 years.

**TABLE 6:** Title of the 25 references with strong citation bursts of PCD in OP.

Rank	Strength	Title
1	14.31	Inhibition of osteoblastogenesis and promotion of apoptosis of osteoblasts and osteocytes by glucocorticoids—Potential mechanisms of their deleterious effects on bone^25^
2	13.53	Increased bone formation by prevention of osteoblast apoptosis with parathyroid hormone^33^
3	14.3	Birth and death of bone cells: Basic regulatory mechanisms and implications for the pathogenesis and treatment of osteoporosis^27^
4	13.17	Promotion of osteoclast survival and antagonism of bisphosphonate‐induced osteoclast apoptosis by glucocorticoids^36^
5	14.13	Glucocorticoids act directly on osteoblasts and osteocytes to induce their apoptosis and reduce bone formation and strength^28^
6	13.3	The Amazing Osteocyte^42^
7	15.19	From Estrogen‐Centric to Aging and Oxidative Stress: A Revised Perspective of the Pathogenesis of Osteoporosis^30^
8	12.66	Matrix‐embedded cells control osteoclast formation^45^
9	15.13	Standardized Nomenclature, Symbols, and Units for Bone Histomorphometry: A 2012 Update of the Report of the ASBMR Histomorphometry Nomenclature Committee^46^
10	14.54	Prevention of glucocorticoid induced‐apoptosis of osteoblasts and osteocytes by protecting against endoplasmic reticulum (ER) stress *in vitro* and *in vivo* in female mice^47^
11	14.74	Autophagy in osteoblasts is involved in mineralization and bone homeostasis^34^
12	17.12	Oxidative stress in bone remodeling: Role of antioxidants^48^
13	16.19	Autophagy controls mesenchymal stem cell properties and senescence during bone aging^49^
14	16.19	Defective autophagy in osteoblasts induces endoplasmic reticulum stress and causes remarkable bone loss^50^
15	15.15	Autophagy Maintains the Function of Bone Marrow Mesenchymal Stem Cells to Prevent Estrogen Deficiency‐Induced Osteoporosis^51^
16	13.67	An overview and management of osteoporosis^52^
17	13.67	Autophagy as a target for glucocorticoid‐induced osteoporosis therapy^53^
18	13.3	Inactivation of autophagy ameliorates glucocorticoid‐induced and ovariectomy‐induced bone loss^54^
19	13.18	Osteoporosis treatment: Recent developments and ongoing challenges^55^
20	12.75	Postmenopausal Osteoporosis^56^
21	27.85	Osteoporosis^57^
22	15.18	Osteoblast–osteoclast interactions^58^
23	22.52	Autophagy in bone homeostasis and the onset of osteoporosis^59^
24	12.46	Reactive Oxygen Species in Osteoclast Differentiation and Possible Pharmaceutical Targets of ROS‐Mediated Osteoclast Diseases^60^
25	12.46	Targeting autophagy in osteoporosis: From pathophysiology to potential therapy^61^

### Analysis of Keywords

Through the co‐occurrence analysis of keywords, we could quickly capture research hot spots in a certain field. There are 5191 author keywords on research on PCD in OP over the past two decades. Table 7 shows the top 40 high‐frequency keywords in research on PCD in OP. Among these keywords, osteoporosis, apoptosis, osteoblast, osteoclast, autophagy, osteoblasts, oxidative stress, bone appeared more than 100 times, and they represented the main research directions of PCD in OP.

**TABLE 7:** Top 40 keywords on research of PCD in OP.

Rank	Keywords	Counts	Rank	Keywords	Counts
1	Osteoporosis	913	21	Bisphosphonates	59
2	Apoptosis	444	22	Aging	55
3	Osteoblast	275	23	Osteoarthritis	55
4	Osteoclast	230	24	Dexamethasone	54
5	Autophagy	219	25	Osteocytes	54
6	Osteoblasts	166	26	Postmenopausal osteoporosis	53
7	Oxidative stress	142	27	Bisphosphonate	51
8	Bone	102	28	Ferroptosis	44
9	Osteoclasts	93	29	Bone mineral density	43
10	Osteogenesis	75	30	Bone remodeling	43
11	Proliferation	74	31	Estrogen	43
12	Osteogenic differentiation	72	32	Bone formation	42
13	Inflammation	71	33	Cancer	42
14	Osteoclastogenesis	69	34	Reactive oxygen species	42
15	Glucocorticoids	66	35	Glucocorticoid‐induced osteoporosis	40
16	Osteocyte	66	36	Mesenchymal stem cells	40
17	Differentiation	63	37	Bone marrow mesenchymal stem cells	38
18	Glucocorticoid	63	38	Ovariectomy	35
19	Rankl	63	39	Osteoprotegerin	34
20	Bone resorption	61	40	NF‐kappa B	32

**FIGURE 11:**
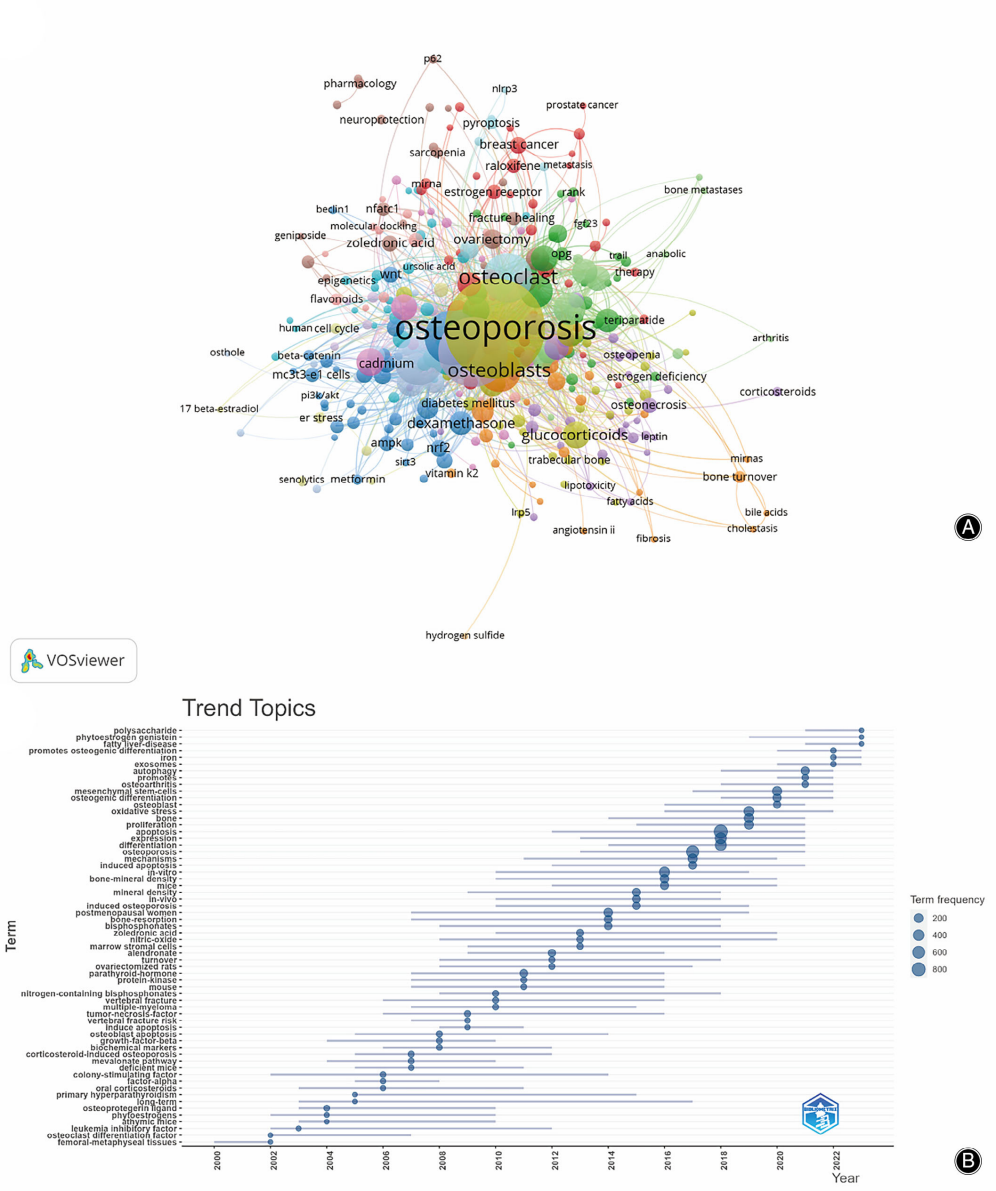
Keyword cluster analysis (A) and Trend topic analysis (B) of PCD in OP.

We filtered keywords with numerous occurrences greater than or equal to five and performed cluster analysis through VOSviewer (Figure 11A). As shown in Figure 11A, we obtained 16 clusters in total, representing 16 research directions. The keywords in the yellow clusters consist of osteoporosis, osteocyte, rat, histomorphometry, endoplasmic reticulum stress, angiogenesis, etc. The keywords in the blue clusters consist of melatonin, oxidative damage, osteoblast, reactive oxygen species, etc. The keywords in the green clusters consist of bone, bone remodeling, cytokines, osteoblastogenesis, osteoclastogenesis, osteoprotegerin, etc.

The analysis of the topic trends of the keywords (Figure 11B) showed that from 2000 to 2010, the research in this period mainly focused on cytokines, zoopery, osteoprotegerin ligand, and the main keywords were tumor‐necrosis‐factor, growth‐factor‐beta, colony‐stimulating factor, deficient mice, athymic mice, osteoblast apoptosis. Since 2010, scholars have been actively exploring bone cells and the pathogenesis of PCD in OP, and the main keywords are apoptosis, expression, bone, mechanisms, mesenchymal stem‐cells, autophagy, osteoblast, etc.

## Discussion

### General Information

In this study, the literature in the field of PCD in OP was analyzed qualitatively and quantitatively using bibliometric analysis software such as CiteSpace, VOSviewer, and the “bibliometrix” R package, and the research results and progress were reviewed. Basic information such as number of publications per year, countries, authors, institutions, references, journals, keywords, were analyzed qualitatively and quantitatively. The total number of papers published in the field since 2000 is 1394 with an overall increasing trend. This study divides time into three phases based on the rate of increase in the number of publications per year. Between 2000 and 2012, the annual average number of publications in this field was about 60, indicating that PCD in OP has begun to receive attention from scholars, and research on PCD in OP was in its infancy during this period. Between 2013 and 2017, the field published an average of approximately 120 papers per year. The number of publications began to increase significantly during this time, with scholars emphasizing research in OP and PCD. Between 2018 and 2023, there is a significant increase in publications, averaging around 240 per year, indicating that research on PCD in OP is in a period of explosion.

China and the United States are the primary countries researching PCD in OP. China ranks first in the world in terms of the number of publications in the literature, well ahead of the United States, which ranks second. In terms of citations of published literature, the United States ranks first in the world in terms of the quality of published literature, and China ranks second. It is evident that research on PCD in OP is more advanced in the United States, and with the gradual increase in China's scientific research funding, the quality of China's research is catching up and improving, and is gradually converging with international publications on OP research. It is worth noting the close cooperation between China, the United States, Japan, and Canada. There is active cooperation between the United Kingdom and the United States and Germany, as well as close cooperation between Italy and the United States. Furthermore, developed countries, such as the United States, Japan, and the United Kingdom (Figure 2), have invested in this research area earlier, with a primary focus on the period before 2016. This trend may be closely related to the earlier entry of developed countries into an aging society. Developing countries represented by China, India, etc. are also gradually moving towards an aging society due to their large population base. Therefore, since 2016, research on OP and PCD has become the focus of these developing countries, which have become an emerging force in this research field.

Nine of the top 10 institutions are located in China and the other one is located in the United States. We found that Shanghai Jiao Tong University (SJTU) published the highest number of papers. Meanwhile, SJTU has close cooperation with Fudan University, Tongji University, Chinese Academy of Sciences, Second Military Medical University, and Zhejiang University. Cooperation between research institutions is beneficial for the long‐term development of academic research. Although cooperative relationships exist between institutions in different countries, the breadth and intensity of cooperation between these institutions is not ideal. For instance, there is little cooperation among institutions in the United States, China, and Canada. In the long run, this situation will not promote the development and progress of the research field. Therefore, we strongly recommend that extensive cooperation and exchanges between research organizations in various countries be actively pursued in order to jointly promote in‐depth research on PCD in OP.^62^

The largest number of studies of PCD in OP were published in *Bone* (IF = 4.1, Q1), which shows that *Bone* is currently the most popular journal in this research area. The journal with the highest impact factor was *Biomedicine & Pharmacotherapy* (IF = 7.5), followed by *Journal of Bone and Mineral Research* (IF = 6.2) and *International Journal of Molecular Sciences* (IF = 5.6). For the cocited journals, we found that most of them were high‐impact Q1 and Q2 journals in the JCR. It is clear that these journals are high‐quality international journals that support research on PCD in OP. Additionally, research on PCD in OP has primarily been published in molecular, biological, and immunological journals and pharmaceutical, medical, and clinical journals. This suggests that the field has reached a mature stage of development, with research covering both basic experimental and clinical treatments.

The most published authors are Liu Zong‐Ping, Yang Lei, and Stavros C. Manolagas, each with over eight papers. In terms of co‐cited authors, Ronald S. Weinstein is the most co‐cited author with 743 citations, followed by Stavros C. Manolagas with 521 citations, and Robert L. Jilka with 382 citations. According to the WOScc database search, we found that the number of published articles of Robert S. Weinstein is 391, and his main research direction is the effect of apoptosis mechanism of bone tissue cells on osteoporosis. Obviously, the findings of Robert S. Weinstein laid the theoretical and experimental foundation for the study of PCD in OP.

A co‐cited reference is one that has been cited by multiple other documents and is therefore considered a foundational piece of research in a particular field. In this study, we selected 20 of the most popular studies to determine the research base of PCD in OP. Among them, the five most popular documents were co‐cited more than 125 times in total. Weinstein et al. 1998 “Inhibition of osteoblast ogenesis and promotion of apoptosis of osteoblasts and osteocytes by glucocorticoids—Potential mechanisms of their deleterious effects on bone” is the most cited study.^25^ The study discovered that long‐term administration of glucocorticoids (GCs) affects bone by inhibiting osteoblast and osteoclast precursors and promoting apoptosis of mature osteoblasts and osteocytes in mice. William J. Boyle published the second ranked co‐cited paper in 2003, which delved into the activation of the RANKL signaling pathway during osteoclast formation, the mechanism of bone resorption, and the effect of hormones on bone structure, providing a molecular basis for further research on the treatment of osteoporosis. Stavros C. Manolagas published the third most cited article in 2000, which provides a detailed review of the mechanisms that regulate osteoclast and osteoblast production and apoptosis, and their significance for the pathogenesis and treatment of osteoporosis. Charles A. O'Brien published the fourth ranked co‐cited article in 2004, which found that osteoblasts and osteoclasts are the direct targets of GCs in vivo, and that excessive GCs induce apoptosis of osteoblasts and osteoclasts, which reduces bone formation and bone strength. Lilian I. Plotkin published the fifth most cited article in 1999, which showed experimentally that bisphosphonates and calcitonin could have a therapeutic effect on glucocorticoid‐induced osteoporosis by organizing apoptosis of osteoblasts and osteoclasts. Undoubtedly, these papers have laid the foundation for PCD research in the field of OP.

### Hotspots and Frontiers

In terms of the references with citation bursts, we found that the effect of GCs on the apoptotic mechanism of bone tissue cells was the main research content of the early strong citation burst references related to PCD in OP.^25^ Apoptosis is the physiological death of cells, and plays an important role in stabilizing the internal environment of tissues. Osteoblasts are the main target of glucocorticoid action and play an important role in the pathogenesis of glucocorticoid‐induced osteoporosis (GIOP). Long‐term administration of GCs leads to overexpression of ROS, which induces osteoclast apoptosis via the PKCβ/p66(she)/JNK pathway, while excessive GCs also activate caspase activity and inhibit the Wnt/β‐catenin signaling pathway, which is important for osteoclastogenesis and bone metabolism, to induce and promote osteoclast apoptosis.^63^ In addition the main genes involved in osteoblast apoptosis in GIOP are Bcl‐2, p53, Fas, and Fasl genes. In conclusion, GCs induce and promote apoptosis in outcome cells through various mechanisms that contribute to the development of GIOP.^64^

However, since 2019, research has focused on the potential therapeutic mechanisms of autophagy in osteoporosis and the pathogenic mechanisms of oxidative stress in osteoporosis. In addition to citation bursts, keywords can also help us quickly capture the distribution and evolution of hotspots in the PCD field in OP. Table 6 includes keywords such as glucocorticoids, autophagy, mesenchymal stem cells, osteogenic differentiation, oxidative stress, and iron death in addition to the main keywords such as osteoporosis, osteoblasts, osteoclasts, and apoptosis. Based on the analysis of the trending themes of the keywords, it was found that the early studies focused on the effects of cytokines and GCs of osteoporosis and the studies were mostly animal experiments. Much of the research in recent years has focused on the effects of apoptosis, autophagy, and oxidative stress regulating osteoblasts and osteoclasts on osteoporosis. The analysis of keywords can provide valuable insights into the research hotspots, development directions, and clinical significance of keywords in the field of PCD research in OP. These findings can enable researchers to align their research directions with trends in research development and deepen the study of the pathogenesis of OP.

Based on keyword clustering analysis and trend theme analysis, we concluded that oxidative stress and autophagy are the current hotspots for PCD research in OP. Out of 2905 publications, there were a total of 551 studies on oxidative stress, with no more than 10 publications per year before 2011 and more than 50 publications on oxidative stress since 2019. There are a total of 456 studies on autophagy, and no studies on autophagy were conducted before 2008. The number of papers published during the 10‐year period of 2008–2017 was relatively small, all less than 20. Since 2018, studies have significantly increased, with an average of around 50 publications per year. Autophagy has been emphasized by scholars due to the Nobel Prize in Physiology or Medicine, which was awarded to Japanese scientist Ryoori Ohsumi for his discoveries on cellular autophagy mechanisms in 2016. This emphasis may explain the rapid increase in the number of its studies in recent years. Thus, oxidative stress and autophagy may be the current research hotspot in the field of PCD in OP.

**FIGURE 12:**
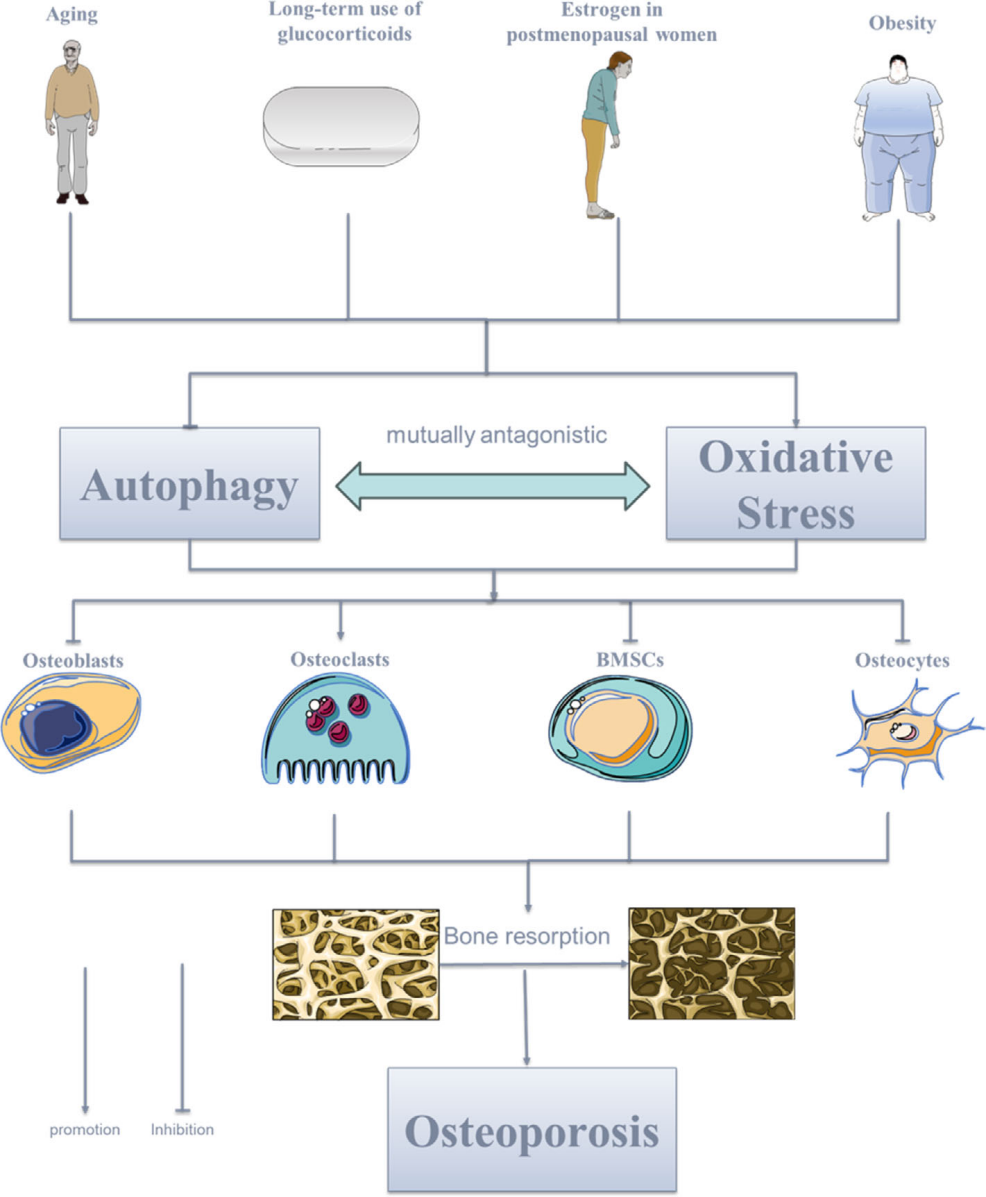
Mechanism of autophagy and oxidative stress on osteoporosis.

Under the influence of aging,^65^ long‐term glucocorticoid effect,^66^ decrease of estrogen in menopausal women,^67^ obesity,^68^ the up‐regulation of oxidative stress and down‐regulation of autophagy lead to the apoptosis of osteoblasts and osteocytes, the decrease of differentiation of bone marrow mesenchymal stem cells (BMSCs), and the enhancement of osteoclasts' activity, which lead to the decrease of bone mass and finally the formation of osteoporosis. In vitro studies have shown that autophagy is negatively correlated with oxidative stress, upregulation of cellular autophagy is associated with reduced oxidative stress, and downregulation of autophagy leads to increased oxidative stress in osteoblast‐like cells^69^ (Figure 12).

Oxidative stress refers to an imbalance between the body's oxidative and antioxidant effects caused by an excess of free radicals, including reactive oxygen species (ROS) and reactive nitrogen species (RNS).^70^ This imbalance can lead to cellular damage. Oxidative stress induced by ROS is prevalent in populations with a high risk of osteoporosis, including postmenopausal women, older men, long‐term users of GCs, and obese individuals.^71^ ROS initiates the mitochondrial apoptosis signaling pathway to induce increased expression of osteoclast markers such as c‐Fos, NFATc1, TRAP, which attenuates osteogenesis, leading to an imbalance in bone reconstruction and causing bone loss.^72^ Antioxidant enzymes help to neutralize the harmful effects of ROS and also play a role in regulating bone metabolism. Glutathione peroxidase is an enzyme that reduces organic hydroperoxides and H_2_O_2_ by using glutathione. This process helps to prevent oxidative damage and contributes to the stabilization of bone tissue.^73^, ^74^, ^75^ Peroxiredoxin (PRDX), as an antioxidant enzyme, plays an important role in the development of osteoporosis.^76^ Research has demonstrated that osteoporosis can be prevented by reducing oxidative stress in osteoclasts through antioxidant supplementation.^77^

Autophagy is biologically a catabolic pathway that is essential for cell survival and plays a key role in maintaining cellular and tissue homeostasis.^78^ Autophagy is classified into three categories: molecular chaperone autophagy, microautophagy, and macroautophagy. Molecular chaperone autophagy is the direct transport of substances from the cytoplasm into the lysosome mediated by molecular chaperone proteins.^79^ Microautophagy is the direct capture of small amounts of nearby cytoplasm by forming depressions or protrusions in the lysosomal membrane.^80^ In macroautophagy, the formation of autophagosomes symbolizes the capture and delivery of intracellular substances.^81^ Of these three types of autophagy, macroautophagy is most closely related to cell biology, physiology, and disease, and is generally referred to as “autophagy.” Increasing evidence suggests that there is an important correlation between autophagy and bone homeostasis, which is mediated by bone marrow mesenchymal stem cells, osteoblasts, osteocytes, and osteoclasts.^53^ In BMSCs, inhibition of autophagy results in the accumulation of ROS and DNA damage. Autophagy is triggered by a large amount of oxidative stress, and its antagonism against ROS serves to protect BMSCs to a certain extent.^61^ In osteoblasts, autophagy protects osteoblasts from cytotoxic stimuli by degrading damaged organelles, and in vitro studies have shown that autophagy can antagonize oxidative stress‐mediated apoptosis in osteoblasts.^82^ osteocyte, as long‐lived cells, utilize autophagy to cope with bone loss and bone homeostasis imbalance caused by unfavorable factors such as high ROS and hypoxia, which is a survival mechanism of osteoblasts in response to stress.^83^ Autophagy is also actively involved in osteoclast differentiation. Autophagy is activated and promotes osteoclast differentiation during RANKL‐stimulated osteoclast differentiation.^83^

New studies are constantly emerging in the field of PCD in OP. However, some research hotspots may have been overlooked due to the short publication time.^84^ Out of 2905 publications, we found a total of 68 studies on ferroptosis, no studies on ferroptosis until 2019, and an average of no more than five studies published per year from 2019 to 2021, but a surge in publications to 30 in 2022. The total number of studies on pyroptosis is 31, which has only been studied since 2020 and is increasing year by year. In 2023, only three studies on cuproptosis were published. This suggests that ferroptosis, pyroptosis, and cuproptosis may become hot spots for the research on PCD in OP in the future.

## Strengths and Limitations

This study analyzed the knowledge base and research hotspots of research on PCD in OP based on bibliometric tools such as CiteSpace, VOSviewer, bibliometrix R package, and online bibliometric analysis platform. However, this study has some limitations. This study only analyzed the literature on OP and PCD studies in the WoSCC database, while ignoring other databases. Secondly, because new research is updated daily, some highly cited studies published in recent years may be overlooked because of the short publication time. Thirdly, we searched for literature published in English and may have overlooked high‐quality literature in the field of OP and PCD in other languages.

## Conclusion

In summary, this study is the first to use a bibliometric approach to analyze the global research trends in the intersection of PCD and osteoporosis over the past 20 years in a scientifically rigorous and comprehensive manner. This paper has significant research value and potential applications in PCD and OP. The annual rise in the number of papers suggests that scholars worldwide are increasingly emphasizing the research on PCD in OP. The countries involved in this study are mainly China and the United States. Currently, China and the United States are leading the research on PCD in OP. However, there is still a need to strengthen cooperation and communication between countries and agencies. In addition, this study found that oxidative stress and autophagy are the research hotspots of PCD in OP in recent years. However, with the advancement of apoptosis research, including the emergence of processes such as pyroptosis, ferroptosis, and cuproptosis, it is anticipated that there will be further opportunities for research in this field.

## Author Contributions

Author contributions All authors made a significant contribution to the work reported and agreed to be accountable for all aspects of the work. R.Y.F. and L.G. designed the experiments. R.Y.F., L.X.Z, J.K. carried out data extraction. R.Y.F., J.H.F., and L.H.Z. carried out mapping and tabulation. R.Y.F., L.B.W., and L.X.Z prepared the initial draft of the manuscript. L.G. gave critical feedback during the study or during the submission of the manuscript. All authors provided final approval of the version to be submitted and agreed on the journal for publication.

## Conflict of Interest Statement

The authors declare no conflicts of interest for this research.

## Ethics Statement

All data used in this work are publicly available from studies with relevant participant consent and ethical approval.

## Data Availability

Publicly available datasets were analyzed in this study. The Web of Science Core Collection database can be found at the following URL: https://www.webofscience.com/wos/woscc/basic-search.
